# A sequence analysis of hospitalization patterns and service utilization in patients with major psychiatric disorders in China

**DOI:** 10.1186/s12888-021-03251-w

**Published:** 2021-05-11

**Authors:** Xueyan Han, Feng Jiang, Jack Needleman, Moning Guo, Yin Chen, Huixuan Zhou, Yuanli Liu, Chen Yao, Yilang Tang

**Affiliations:** 1 Peking University First Hospital, 8 Xishiku Road, Xicheng District, Beijing, China; 2 Institute of Health Yangtze River Delta, Shanghai Jiao Tong University, 1954 Huashan Road, Xuhui District, Shanghai, China; 3 Department of Health Policy and Management, UCLA Fielding School of Public Health, 650 Charles Young Dr. S., 31-269 CHS Box, Los Angeles, CA 951772 USA; 4 Beijing Municipal Health Commission Information Centre, 277 Zhao Deng Yu Road, Xicheng District, Beijing, China; 5 Peking University International Hospital, 29 Sheng Ming Yuan Road, Haidian District, Beijing, China; 6 School of Sport Science, Beijing Sport University, 48 Xinxi Road, Haidian Street, Beijing, China; 7 School of public health, Chinese Academy of Medical Sciences and Peking Union Medical College, No.3 Dong Dan San Tiao, Dongcheng District, Beijing, China; 8 Peking University Clinical Research Institute, 38 Xueyuan Road, Haidian District, Beijing, China; 9 Department of Psychiatry and Behavioral Sciences, Emory University, 12 Executive Park Drive NE, Suite 300, Atlanta, GA, USA; Atlanta VA Medical Center, 1670 Clairmont Road, Decatur, GA USA; 10 Atlanta VA Medical Center, Decatur, GA USA

**Keywords:** Psychiatric service, Psychiatric readmission, Hospitalization pattern, Sequence analysis, China

## Abstract

**Background:**

Understanding the long-term inpatient service cost and utilization of psychiatric patients may provide insight into service demand for these patients and guide the design of targeted mental health programs. This study assesses 3-year hospitalization patterns and quantifies service utilization intensity of psychiatric patients in Beijing, China.

**Methods:**

We identified patients admitted for one of three major psychiatric disorders (schizophrenia, bipolar and depressive disorders) between January 1 and December 31, 2013 in Beijing, China. Inpatient admissions during the following 3 years were extracted and analyzed using sequence analysis. Clinical characteristics, psychiatric and non-psychiatric service use of included patients were analyzed.

**Results:**

The study included 3443 patients (7657 hospitalizations). The patient hospitalization sequences were grouped into 4 clusters: short stay (*N* = 2741 (79.61% of patients), who had 126,911 or 26.82% of the hospital days within the sample), repeated long stay (*N* = 404 (11.73%), 76,915 (16.26%) days), long-term stay (*N* = 101 (2.93%), 59,909 (12.66%) days) and permanent stay (*N* = 197 (5.72%), 209,402 (44.26%) days). Length and frequency of hospitalization, as well as readmission rates were significantly different across the 4 clusters. Over the 3-year period, hospitalization days per year decreased for patients in the short stay and repeated long stay clusters. Patients with schizophrenia (1705 (49.52%)) had 78.4% of cumulative psychiatric stays, with 11.14% of them in the permanent stay cluster. Among patients with depression, 23.11% had non-psychiatric hospitalizations, and on average 46.65% of their total inpatient expenses were for non-psychiatric care, the highest among three diagnostic groups.

**Conclusion:**

Hospitalization patterns varied significantly among psychiatric patients and across diagnostic categories. The high psychiatric care service use of the long-term and permanent stay patients underlines the need for evidence-based interventions to reduce cost and improve care quality.

**Supplementary Information:**

The online version contains supplementary material available at 10.1186/s12888-021-03251-w.

## Introduction

### Disease burden of mental illnesses

Mental and addictive disorders accounted for 6.8% of global burden of disease (measured by disability-adjusted life years (DALYs)) [1]. Severe psychiatric disorders such as schizophrenia, bipolar disorder, and depressive disorder (especially major depression disorder) were among the leading causes of years lived with disability (YLD) [2, 3] and were recognized as major psychiatric disorders by previous studies [2, 4]. According to the *China Mental Health Survey*, the prevalence of any mental health disorders in China was 9.3%, indicating a high burden of disease [5].

### The importance of understanding inpatient psychiatric care

Understanding the long-term inpatient service cost and utilization of psychiatric patients can quantify the service demand for these patients and guide the design of targeted community-level mental health programs. This is especially the case in low-and-middle-income countries, where a large portion of such needs have yet to be met [6, 7]. Inpatient psychiatric service use need to be assessed as an indicator of the cost burden of these diseases, a proxy for the disease severity [2], and an indicator of the performance of outpatient and community-based systems in meeting patient needs [8].

### The need of analyzing psychiatric hospitalization pattern

Many existing studies on inpatient service utilization of psychiatric patients focus on readmissions over a short follow-up period (e.g. 30 days or 12 months) [9–11]. Studies with extended observation periods often include a small group of patients [12, 13] with a particular diagnosis [14], or are conducted in a single center [15].

There are other studies that included large, multi-centered samples [16–18]; however, these studies often investigate the overall service use and expense in a set timeframe [16, 17, 19] while the long-term trajectory of service utilization, the clustering of service need, the durations between multiple admissions, the demand and cost of general (non-psychiatric) healthcare services are unexamined. Such information can provide insight into the patterns of psychiatric hospitalizations [20, 21] for different psychiatric diagnosis groups and identifying the optimal time points for post-discharge intervention.

### Potential benefits of sequence analysis in service utilization studies

Sequence analysis was originally used to analyze deoxyribonucleic acid (DNA) sequences in biology [20] and then to depict life events sequences in sociology [22]. This method has been utilized to depict long-term inpatient service utilization of psychiatric patients in recent studies [20, 21, 23]. Sequence analysis can be used to track hospitalizations or relapse events of a given group of patients through an extended time period, with the beginning, end and duration of the events and the time gaps between adjacent events being captured and preserved in the analyses [24]. Combined with cluster analysis, previous studies have been able to derive patterns of inpatient service use, which enable the quantification of diversified service needs for psychiatric patients. Since the hospitalization patterns derived from sequence analysis do not rely solely on inpatient expenses or hospitalization frequency [20], its analysis of the timing of repeated admissions can provide insight into service utilization studies.

In a prior study using sequence analysis, Han et al. analyzed discharge medical records of 831 patients with alcohol use disorder (AUD) from a regional database to examine the 3-year hospitalization patterns [20]. They found that a small group of patients (6.61%) in the high-utilizing clusters accounted for 37.26% of total psychiatric hospital bed days in the sample [20]. They also found that approximately 20% of total inpatient expenses for patients in the short-stay cluster were for non-psychiatric inpatient service, the highest among other identified hospitalization patterns [20]. Another study of 892 inpatients from one hospital in Switzerland found that hospitalization trajectories could be split between heavy users (4.9% of patients who had 30.6% of hospital days) and regular users [21]. They also found patients with schizophrenia were overrepresented among heavy users [21].

These studies showed that although psychiatric patients in general and more specifically patients with schizophrenia were likely to be identified as heavy-utilizers [25, 26], further differentiation by long-term service use patterns and diagnoses might enable better identification of patient needs. Furthermore, the hospitalization patterns across different diagnostic groups and different hospitals, as well as the time gaps between admissions warrant further investigation, so that the hospitalization patterns can be better linked to post-discharge care programs.

### About this study

Mental health services in China heavily rely on specialized psychiatric hospitals (which provide nearly 80% of psychiatric services [27]). Overall, community mental health services in China are underdeveloped and need major improvements in both personnel and expertise [28, 29]. Patients tend to crowd into large, tertiary psychiatric hospitals due to the lack of primary mental healthcare and psychiatric rehabilitation facilities [28], which also leads to prolonged inpatient stay (the average length of stay in the mental health hospitals was 50–60 days in 2017) [11]. The top three primary diagnoses in psychiatric inpatient services are schizophrenia and related disorders, bipolar disorder and depression, which accounted for more than 80% of the psychiatric inpatients in China [11, 30]. Researchers [28, 31, 32] and policy makers in China have made efforts to reform the mental health services and inject funding into community mental health services [33] but problems like over-reliance on hospital care [28] and disparities in care and access [15] persist.

This study, utilizing a combination of sequence analysis and cluster analysis, examines hospitalization patterns of patients with the three major psychiatric disorders—schizophrenia and related disorders, bipolar disorder and depression in Beijing, China. The objectives of this study were: 1) to identify and summarize psychiatric hospitalization patterns in patients with 3 major psychiatric disorders; 2) to track the long-term psychiatric and non-psychiatric inpatient service use, expenses and readmission for psychiatric patients across hospitalizations and by diagnoses; 3) to explore the time gaps between multiple admissions of the psychiatric patients.

## Methods

### Data source

The primary data source of the study was the inpatient medical records from 2010 to 2017 in Beijing, China. Anonymized inpatient medical records were obtained from the Beijing Municipal Health Commission Information Center (the Center). The records were standardized [34] to provide information on inpatient socio-demographic characteristics, admission and discharge dates, primary and secondary diagnoses, operations and procedures during the hospital stay, as well as total expenses for each hospitalization. A unique anonymized identification code for each patient allows patient identification across admissions without compromising patient privacy. A team in the Center was responsible for validating the data quality of these records [35].

The inpatient records were collected from 186 (out of 219 (84.93%)) secondary and tertiary hospitals in Beijing. This regional, multi-center database enabled this study to track the patients’ inpatient service use in psychiatric and general hospitals. The 186 hospitals included 10 psychiatric hospitals: all three tertiary psychiatric hospitals and 7 of the 11 secondary psychiatric hospitals in the city [11]. For more details on the hierarchical healthcare systems in China and the psychiatric care services in Beijing city see Additional file 1.

### Study design and ethic approval

This study was designed to identify patients with one of the major psychiatric disorders and follow their subsequent inpatient service use for 3 years. The hospitalization patterns were derived using state-sequence analysis combined with cluster analysis. Stratification analyses were conducted across the identified patterns and patients’ primary diagnosis.

The study protocol was reviewed and approved by the Ethics Committee of Chinese Clinical Trial Registry (ChiECRCT-20,180,166). Informed consent was waived as there was no personal identifiable information in the database. As this study was based entirely on the analysis of routinely collected hospital administrative data, no experiment was conducted. All methods used in this study were in accordance with relevant guidelines and regulations.

### Patient eligibility criteria

Patients included in this study met the following criteria: 1) The patient had been admitted between January 1 to December 31, 2013 with one of the three major psychiatric disorders as the primary diagnosis (schizophrenia and related disorders, bipolar disorder or depression (ICD-10 codes: F20, F31, and F32-F33)); 2) The patient had not been admitted for any psychiatric disorders 3 years prior to their first psychiatric admission in 2013, making the initial 2013 admission an index admission for the study; 3) The index admission was at one of the three tertiary psychiatric hospitals in Beijing; 4) The patient was at least 18 years old on the index admission and had a Beijing residency; 5) The patient did not have an in-hospital death during the 3 year follow up period after the index admission.

We excluded patients with psychiatric admissions within 3 years prior to the index admission to increase homogeneity concerning the disease severity. Patients residing outside of Beijing were excluded because they might have unobserved hospital stays outside the city. We restricted the index admissions in the 3 tertiary psychiatric hospitals [11], which accounted for ~ 80% of the psychiatric inpatients in Beijing city. The restriction on hospital type further increases the homogeneity of the data.

### Patient characteristics and measures of service utilization

#### Patient characteristics

The following patient characteristics were reported: age, gender, insurance status (the Urban Employee Basic Medical Insurance, other insurance types and uninsured), primary discharge diagnosis (the 3 major psychiatric disorders, based on the ICD-10 code), the Charlson Comorbidity Index (CCI) (calculated as the count of comorbidities listed in the CCI, based on the first 10 secondary diagnosis codes in the index admission), discharge hospital of the index admission (the 3 tertiary hospitals were coded as Hospital 1, 2, and 3). Details on the healthcare insurance system in China as well as the complete list of comorbidities in the CCI and its relevance in this study are described in Additional file 1.

#### Measures for service utilization

Admissions were classified as psychiatric and non-psychiatric based on the primary diagnosis of the inpatients (non-psychiatric admissions were primarily admitted for diagnoses codes outside of F00-F99).

Psychiatric service was analyzed in terms of:

- Frequency: Number of admissions; proportion of patients with only one admission);
- Length of stay (LOS, in days): Total psychiatric days in the 3 years of observation as well as the average days in the first, second and third year starting with the index admission for each patient. The cumulative psychiatric hospital days were calculated by adding up the LOS of each patient;
- Hospitalization expenses: Total expenses were estimated and daily expense was calculated for each patient as total psychiatric expenses divided by the total days of psychiatric admissions. Hospital expenses were standardized to 2017 costs using the consumer price index of the healthcare sector in China [36].

For patients with multiple psychiatric admissions during the 3-year period, time gaps between 1st & 2nd, 2nd & 3rd, as well as 3rd &4th psychiatric admission were calculated (if applicable).

We also constructed measures of admission, days and expenses for non-psychiatric admissions, and calculated the proportion of total inpatient expenditures associated with non-psychiatric and psychiatric admissions. The days and expense measures of non-psychiatric service utilization were calculated for those who used non-psychiatric inpatient services in the 3-year observation period.

Psychiatric readmission measures are potential indicators for both service utilization and quality of care. Psychiatric readmission is defined as an admission with a primary diagnosis of any psychiatric disorder in a specific timeframe (30 days or 365 days). Same-day readmissions, readmissions occurring within 24 h of discharge, were common in the study context, and may be a response to hospital or insurance policies rather than true discharges [30]. While many studies exclude same-day readmissions as planned, we included these admissions to flag this phenomenon. Details on data cleansing are discussed further in Additional file 1.

### Statistical analysis

We used state-sequence analysis followed by the cluster analysis in this study. For data transformation, inpatient admission status (admitted or not) of each day in the 3-year observation period was transformed to a sequence of 1095 digits for each patient (0-not admitted; 1-admitted). These sequences were then analyzed using the optimal matching algorithm to produce a dissimilarity matrix. The matrix was then analyzed using cluster analysis (Ward’s method), which grouped patients with similar hospitalization trajectory to the same cluster [24]. In this study, only psychiatric admissions entered the sequence analysis. The non-psychiatric admissions were extracted but not used in the sequence analysis. The sequence analysis was conducted using the TraMineR package and the cluster package in R (version 3.4.4) [24]. Detailed methodology has been reported in previous studies [11, 21]. A detailed account of sequence analysis methods can be found in Additional file 1.

For data visualization, we plotted the patients’ daily admission status over the 1095-day observation period by cluster, with patients in each cluster sorted by total hospitalization days.

For descriptive analyses, we first focused on patient characteristics, the frequency, days and expenses of psychiatric hospitalizations, and psychiatric readmission within and across the clusters. We then conducted stratified analysis according to primary diagnosis, concentrating on the differences in the pattern of care for patients in each diagnostic category. The utilization and expenses of non-psychiatric care among the psychiatric patients were also analyzed.

For statistical tests of group comparisons, we used Kruskal-Wallis tests for continuous variables, and Chi-square tests for categorical variables. Pairwise comparison was conducted if applicable, with Bonferroni correction. The *p*-values reported were two-sided and the significant level was set at *p* < 0.05.

## Results

### Patient characteristics

The study included 3443 eligible patients (approximately 30% of all psychiatric patients of Beijing in 2013), with 7657 hospitalization episodes, 6631 (86.5%) of which were psychiatric admissions. More than half of the included patients (1994/3443, 57.91%) were female, and the mean age was 42.95 (standard deviation (SD): 15.54) years. By primary diagnoses, 1705 (49.52%) had schizophrenia and related disorders, 890 (25.85%) bipolar disorders and 848 (24.63%) depression at the index admission. (Table 1).

**Table 1:** Patient characteristics and service utilization in the 4 hospitalization pattern clusters

	All	Short stay	Repeated long stay	Long-term stay	Permanent stay	Statistics
**Patient characteristics**						
Number of patients (n(%))	3443 (100)	2741 (79.61)	404 (11.73)	101 (2.93)	197 (5.72)	/
Gender: Male (n(%))	1449 (42.09)	1056 (38.53)	198 (49.01)	59 (58.42)	136 (69.04)	χ2(3) = 91.95, *p* < 0.001
Age (mean, SD)	42.95 (15.54)	41.45 (15.31)	44.33 (16.04)	53.11 (11.96)	55.7 (10.48)	H(3) = 216.29, *p* < 0.001
Payment methods (n(%))						χ2(6) = 177.57, *p* < 0.001
Urban employee insurance	1696 (49.26)	1214 (44.29)	236 (58.42)	79 (78.22)	167 (84.77)	
Other insurance	1032 (29.97)	890 (32.47)	101 (25)	15 (14.85)	26 (13.2)	
Un-insured	715 (20.77)	637 (23.24)	67 (16.58)	7 (6.93)	4 (2.03)	
Diagnosis groups (n(%))						χ2(6) = 401.61, *p* < 0.001
Schizophrenia	1705 (49.52)	1134 (41.37)	289 (71.53)	92 (91.09)	190 (96.45)	
Bipolar disorder	890 (25.85)	803 (29.3)	76 (18.81)	6 (5.94)	5 (2.54)	
Depression	848 (24.63)	804 (29.33)	39 (9.65)	3 (2.97)	2 (1.02)	
Charlson Comorbidity Index (at index admission) (n(%))						χ2(6) = 113.63, *p* < 0.001
0	2866 (83.24)	2364 (86.25)	312 (77.23)	64 (63.37)	126 (63.96)	
1	467 (13.56)	313 (11.42)	70 (17.33)	29 (28.71)	55 (27.92)	
> 1	110 (3.19)	64 (2.33)	22 (5.45)	8 (7.92)	16 (8.12)	
Hospitals (n(%))						χ2(6) = 823.45, *p* < 0.001
Hospital 1	1471 (42.72)	1307 (47.68)	150 (37.13)	11 (10.89)	3 (1.52)	
Hospital 2	1041 (30.24)	551 (20.1)	209 (51.73)	87 (86.14)	194 (98.48)	
Hospital 3	931 (27.04)	883 (32.21)	45 (11.14)	3 (2.97)	0 (0)	
**Psychiatric service utilization**						
Single admission (n(%))	2169 (63.00)	2073 (75.63)	96 (23.76)	1 (0.99)	0 (0.00)	χ2(3) = 961.77, *p* < 0.001
Admission frequency (mean (SD))	1.93 (1.82)	1.32 (0.65)	2.48 (1.43)	4.66 (1.54)	7.79 (1.67)^a^	H(3) = 1078.37, *p* < 0.001
Length of stay (LOS, in days, mean (SD))						
LOS in the 1st year	76.21 (92.27)	40.48 (22.25)	140.21 (83.21)	236.1 (118.71)	360.18 (25.18)	H(3) = 1388.71, *p* < 0.001
LOS in the 2nd year	32.09 (90.78)	3.02 (10.78)	28.47 (45.55)	195.29 (92.61)	360.29 (24.79)	H(3) = 871.52, *p* < 0.001
LOS in the 3rd year	29.12 (88.51)	2.8 (11.16)	21.71 (40.56)	161.77 (150.62)	342.48 (57.64)	H(3) = 707.04, *p* < 0.001
Total LOS in 3 years	137.42 (253.39)	46.3 (27.95)	190.38 (85.28)	593.16 (107.89)	1062.95 (76.6)	H(3) = 1662.68, *p* < 0.001
Cumulative psychiatric hospital days (n(%))	473,137 (100)	126,911 (26.82)	76,915 (16.26)	59,909 (12.66)	209,402 (44.26)	/
Psychiatric hospitalization expense (in 2017 RMB, mean (SD))						
Daily expense^b^	541.69 (181.84)	556.7 (196.66)	462.09 (90.35)	492.18 (76.31)	521.46 (49.14)	H(3) = 169.36, *p* < 0.001
Total expense	70,194.39 (131,864.89)	24,586.9 (15,689.07)	87,743.86 (43,141.05)	293,584.63 (74,912.52)	554,243.83 (65,508.78)	H(3) = 1593.54, *p* < 0.001
Cumulative psychiatric hospital expense (in million RMB) (n(%))	241.68 (100)	67.39 (27.89)	35.45 (14.66)	29.65 (12.27)	109.19 (45.18)	/
**Psychiatric readmission**						
Same-day readmission (n(%))	346 (10.05)	66 (2.41)	53 (13.12)	40 (39.60)	187 (94.92)	χ2(3) = 1800, *p* < 0.001
30-day readmission (n(%))	480 (13.94)	139 (5.07)	107 (26.49)	42 (41.58)	192 (97.46)	χ2(3) = 1400, *p* < 0.001
365-day readmission (n(%))	862 (25.04)	381 (13.90)	217 (53.71)	67 (66.34)	197 (100.00)	χ2(3) = 978.08, *p* < 0.001
**Non-psychiatric service utilization**						
Patients with non-psychiatric admission (n(%))	579 (16.82)	503 (18.35)	54 (13.37)	11 (10.89)	11 (5.58)	χ2(3) = 28.36, *p* < 0.001
Total LOS (in 3 years) (mean (SD))^c^	21.15 (37.82)	21.41 (39.71)	17.59 (15.35)	25.45 (40.67)	22.36 (22.91)	H(3) = 1.20, *p* = 0.75
Daily expense (mean (SD))^bc^	1768.29 (2584.86)	1793.95 (2666.45)	1557.12 (2232.81)	1647.81 (1169.43)	1751.99 (1091.22)	H(3) = 4.37, *p* = 0.22
Total expense (in 3 years) (mean (SD))^c^	29,614.47 (45,746.36)	30,086.12 (47,101.62)	23,005.42 (33,434.24)	29,057.69 (35,209.69)	41,048.84 (45,081.99)	H(3) = 1.55, *p* = 0.67
Proportion of non-psychiatric expense/the total expense (mean (SD)) ^dc^	40.66%(26.58%)	44.35%(26.05%)	19.34%(14.90%)	10.47%(11.39%)	6.53%(6.57%)	H(3) = 89.96, *p* < 0.001

^a^The admissions in permanent stay cluster were almost all same-day readmissions (mostly planned and for administrative reasons) since the patients in this cluster tended to stay permanently in the hospital (Fig. 1)

^b^The daily expense was calculated for each patient as total psychiatric/non-psychiatric expenses of 3 years divided by the total psychiatric/non-psychiatric hospital days

^c^This indicator was calculated for patients with non-psychiatric admission in the 3-year observation period

^d^The proportion of the non-psychiatric expense in the total expense was calculated as total non-psychiatric expense/(total psychiatric expense + total non-psychiatric expense)*100%

### Characteristics of the identified clusters

Based on the sequence analysis and cluster analysis, we classified the patient hospitalization sequences into four clusters: short stay (*n* = 2741, 79.61%), repeated long stay (*n* = 404, 11.73%), long-term stay (*n* = 101, 2.93%) and permanent stay (*n* = 197, 5.72%).

Most patients, regardless of their diagnoses, were in the short stay cluster, including > 90% of patients with bipolar disorder and depression, and 66.51% of patients with schizophrenia. The long-term stay and permanent stay clusters were composed principally of schizophrenia patients. The long-term stay and permanent stay clusters had fewer uninsured patients and more elder patients with physical comorbidities (Table 1). More than 80% of the patients in the long-term stay and permanent stay cluster were initially hospitalized in Hospital 2.

### Psychiatric service utilization, expense and readmissions across the clusters

Nearly two-thirds of patients, 2169 (63.00%), had only one observed hospitalization in the 3-year observation period. For patients grouped in the short stay cluster, this was three-quarters (75.63%). Only 8.65% of patients were classified into the long-term stay and permanent stay clusters, but these patients accounted for 56.92% of cumulative hospital days. All the patients in the permanent cluster had at least one readmission, and on average had 7.79 admissions, of which most were same day readmissions possibly driven by hospital or insurance policies.

**Fig. 1:**
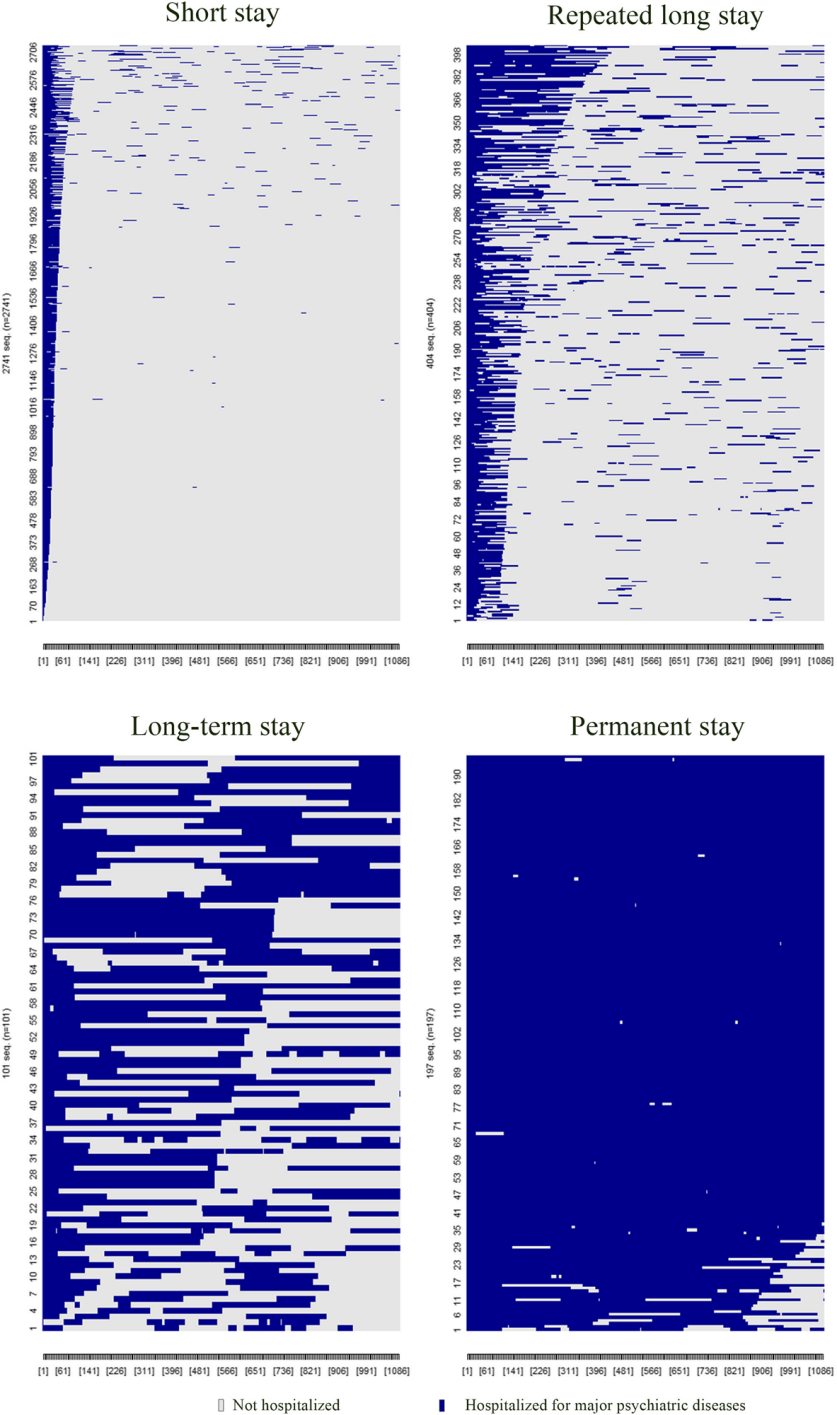
Hospitalization trajectories for patients in the 4 hospitalization pattern clusters. For patients in each of the 4 identified hospitalization pattern clusters, their hospitalization status (either hospitalized (in blue) or not hospitalized (in grey)) for each of the 1095 days of observation (starting from the patient index admission) are illustrated. The figure presents inpatient service utilization for patients in each identified cluster on a time axis (from Day 0 (index admission) to Day 1095 (end of observation)). It highlights variations in hospitalization patterns across patients and clusters through contrasting-colored blocks for days when hospitalized or not for a primary psychiatric diagnosis

Across all clusters, hospitalized days per year declined over the three-year period, easily observable by the increased grey on the right half of each panel in Fig. 1. For patients in the short stay cluster, many of whom had only a single admission, hospitalized days on average dropped from 40.48 days in the first year to about 3 days in the second year (Table 1).

Total expenses per patient over the entire 3-year period were slightly above 70,000 RMB (about 10,000 US dollars). The highest spending was for patients in the permanent stay category (over 550,000 RMB), then long-term stay, repeated long-stay and finally short stay patients (slightly less than 25,000 RMB). The patients in the long-term stay and permanent stay clusters accounted for 57.45% of cumulative psychiatric inpatient expenses. Average daily expense, however, was significantly higher for patients in the short stay cluster (556.7 RMB, about 80 US dollars) with average daily expense in the other three clusters ranging from 462 to 521 RMB (Table 1).

The same-day, 30-day and 365-day readmission rates varied significantly across 4 clusters. The 365-day readmission rates ranged from 13.90% in the short stay cluster to 100% in the permanent stay cluster. With permanent stay patients hospitalized on average for 1063 of 1095 days of the three-year period, patients were rapidly cycled in and out of the hospital for administrative or insurance reasons, with nearly all (94.92%) readmitted on the same day of discharge (Table 1).

### Patient characteristics in different diagnostic categories

Socio-demographic characteristics differed significantly among patients with the 3 major psychiatric disorders. Patients with depression were more likely to be female, older and had health insurance. Patients with schizophrenia were more likely to be male. Patients with bipolar disorder were the least likely to have insurance. The Charlson comorbidity index was not significantly different among patients with different diagnoses at the index admission (Table 2), with over 80% of the patients having no Charlson comorbidities at baseline.

**Table 2:** Patient characteristics and service utilization in the 3 major psychiatric disease categories

	Schizophrenia	Bipolar disorder	Depression	Statistics
**Patient characteristics**				
Number of patients (n(%))	1705 (49.52)	890 (25.85)	848 (24.63)	/
Gender: Male (n(%))	805 (47.21)	355 (39.89)	289 (34.08)	χ2(2) = 42.46, *p* < 0.001
Age (mean, SD)	41.09 (14.86)	40.76 (14.97)	48.99 (15.91)	H(2) = 157.72, *p* < 0.001
Payment methods (n(%))				χ2(4) = 44.66, *p* < 0.001
Urban employee insurance	849 (49.79)	424 (47.64)	423 (49.88)	
Other insurance	526 (30.85)	219 (24.61)	287 (33.84)	
Un-insured	330 (19.35)	247 (27.75)	138 (16.27)	
Charlson Comorbidity Index (at index admission) (n(%))				χ2(4) = 8.75, *p* = 0.07
0	1396 (81.88)	752 (84.49)	718 (84.67)	
1	257 (15.07)	113 (12.7)	97 (11.44)	
> 1	52 (3.05)	25 (2.81)	33 (3.89)	
**Psychiatric service utilization**				
Single admission (n(%))	964 (56.54)	559 (62.81)	646 (76.18)	χ2(2) = 93.73, *p* < 0.001
Admission frequency (mean (SD))	2.36 (2.29)	1.62 (1.14)	1.38 (0.91)	H(2) = 98.26, *p* < 0.001
Total length of stay in 3 years (in days, mean (SD))	217.55 (330.85)	67.26 (98.48)	49.94 (69.09)	H(2) = 377.68, *p* < 0.001
Cumulative psychiatric hospital days (n(%))	370,928 (78.40)	59,857 (12.65)	42,347 (8.95)	/
Total expense (mean (SD))	110,305.81 (172,878.59)	34,296.48 (51,565.11)	27,221.73 (36,092.11)	H(2) = 307.21, *p* < 0.001
Daily expense (mean (SD)) ^a^	520.53 (155.72)	543.24 (176.39)	582.59 (224.41)	H(2) = 78.86, *p* < 0.001
Cumulative psychiatric hospital expense (in million RMB)	188.07 (77.82)	30.52 (12.63)	23.08 (9.55)	/
**Psychiatric readmission**				
Same-day readmission (n(%))	301 (17.65)	24 (2.7)	21 (2.48)	χ2(2) = 216.11, *p* < 0.001
30-day readmission (n(%))	362 (21.23)	53 (5.96)	65 (7.67)	χ2(2) = 150.68, *p* < 0.001
365-day readmission (n(%))	550 (32.26)	164 (18.43)	148 (17.45)	χ2(2) = 94.08, *p* < 0.001
**Non-psychiatric service utilization**				
Patients with non-psychiatric admission (n(%))	227 (13.31)	156 (17.53)	196 (23.11)	χ2(2) = 39.31, *p* < 0.001
Total length of stay in 3 years (in days, mean (SD))	18.47 (28.64)	23.76 (48.9)	22.17 (37.08)	H(2) = 4.39, *p* = 0.11
Daily expense (mean (SD)) ^ab^	1569.43 (1605.57)	1720.87 (1970.09)	2036.34 (3689.43)	H(2) = 0.67, *p* = 0.72
Total expense (in 3 years) (mean (SD))^b^	24,921.58 (36,182.94)	31,942.02 (55,886.31)	33,197.07 (46,478.5)	H(2) = 5.61, *p* = 0.06
Proportion of non-psychiatric expense/the total expense (mean (SD)) ^cb^	34.47%(25.22%)	42.13%(25.40%)	46.65%(27.61%)	H(2) = 23.05, *p* < 0.001

^a^The daily expense was calculated for each patient as total psychiatric/non-psychiatric expenses of 3 years divided by the total psychiatric/non-psychiatric hospital days

^b^The denominator for the mean was patients with non-psychiatric admission in the 3-year observation period

^c^The proportion of the non-psychiatric expense in the total expense was calculated as total non-psychiatric expense/(total psychiatric expense + total non-psychiatric expense)*100%

### Psychiatric care utilization, expenses and readmissions across diagnostic categories

Patients with depression utilized the least amount of psychiatric inpatient care: 76.18% of them had only 1 admission and an average of 49.94 days in the hospital over the three-year period. Mean daily expenses of the depression patients, however, were the highest in the 3 diagnosis groups. As for the cumulative resource utilization, patients with schizophrenia (49.52% in the whole sample) accounted for 78.4% of cumulative hospital days, and consumed 77.82% of cumulative psychiatric inpatient expenses (Table 2).

The 30-day and 365-day readmission rates varied significantly between patients with schizophrenia and patients with bipolar disorder or depression. The 30-day readmission rate for schizophrenia patients was 21.23%, about 3 times as high as the rate for patients with depression (7.67%).

For those with multiple hospitalizations, the gaps between admissions shortened over time. This trend was particularly pronounced for patients with bipolar disorders (Table 3), among whom the median days between the 1st & 2nd admission was 368 days (applicable to 331 patients), and then 225 days (applicable to 113 patients) between the 2nd & 3rd admission, 53 days (applicable to 45 patients) for the 3rd & 4th admission.

**Table 3:** Days between admissions, by primary diagnoses

	Schizophrenia	Bipolar disorder	Depression
Days between 1st & 2nd admission^a^ (Median, interquartile range (IQR))	40, 0–372 (*n* = 741)	368, 105–640 (*n* = 331)	131, 14–381 (*n* = 202)
Days between 2nd & 3rd admission (Median, IQR)	0, 0–117 (*n* = 437)	225, 41–427 (*n* = 113)	79, 11–262 (*n* = 66)
Days between 3rd & 4th admission (Median, IQR)	0, 0–0 (*n* = 305)	53, 4–166 (*n* = 45)	144, 15–255 (*n* = 24)

^a^ The time gaps between multiple admissions were measured in days and reported as median, 25% percentile-75% percentile. The applicable sample size for each cell is presented in parenthesis

### Non-psychiatric care utilization

We found that 16.82% of the included patients had non-psychiatric admissions and the average daily expense of the non-psychiatric hospitalization was about 2000 RMB (about 300 US dollars), quadruple that of a psychiatric admission (~ 500 RMB, about 70 US dollars) (Table 1). Total non-psychiatric care expenses were not significantly different across 4 clusters nor diagnostic categories, but given the lower spending on psychiatric care for patients in the short stay cluster, the proportion of total expenses on non-psychiatric admissions for this group, at 44.35%, was the highest (Table 1). Nearly a quarter (23.11%) of patients with depression had non-psychiatric admissions. The spending on these admissions accounting for 46.65% of their total inpatient expenses, highest among the 3 diagnosis groups (Table 2).

## Discussion

### Main findings

This study utilized the sequence analysis to characterize the psychiatric hospitalization trajectory, resource utilization, and total expenses for patients with 3 major psychiatric disorders in Beijing, China. We found four distinct admission patterns among these patients: short stay (79.61%), repeated long stay (11.73%), long-term stay (2.93%) and permanent stay (5.72%) cluster. The length and frequency of hospitalization, total expenses, 30-day and 365-day psychiatric readmission rates varied significantly across the 4 clusters.

### Hospitalization patterns varied across diagnostic groups

We found that patients with schizophrenia accounted for more than 90% of the long-term stay and permanent stay clusters, which was consistent with findings from Golay et al. [21]. We also found that patients with schizophrenia had much higher readmission rates, hospitalization frequency and length of stay, in line with the finding of other studies [20, 30]. Compared with the sequence analysis results of the patients with AUD in Han et al. [20], this study showed that 5.72% of the patients with major psychiatric disorders (MPD) were grouped into the permanent stay cluster, much higher than the 1.68% of patients with AUD. The mean 3-year total psychiatric hospital days for permanent stay patients with MPD (1062.95 (SD: 76.6) was also much higher than those with AUD in this cluster (818.14 (SD: 225.22)) [20]. These findings highlighted the difference in hospitalization patterns for patients with different psychiatric disorders, and the importance of targeted and tailored interventions for inpatients with varied conditions and severities.

### Implications of variations in hospitalization for the design of interventions

We found that patient age and gender, diagnosis in the index admission, medical comorbidities, payment methods, as well as institutional factors (hospital of the index admission) were associated with variation in patterns of long-term service use. Previous studies have also examined variables including gender, age, primary diagnoses, medical comorbidities and insurance coverage and their association with psychiatric readmissions [11, 37, 38], an important component of long-term service use. Future studies should consider the inclusion of community and home care factors to explain the variation in hospitalization patterns [10, 39, 40].

The fact that the overwhelming majority of patients in the long-term stay and permanent stay clusters were initially hospitalized in Hospital 2, suggests that: i) this hospital may have a different service delivery model compared to other included hospitals, and ii) patients discharged from Hospital 2 may benefit the most from a targeted long-term secondary and community-level psychiatric care programs. Further investigation may need to focus on hospitalization patterns in different hospitals [14, 20] and how characteristics of the hospital may affect the planning of post-discharge care.

A critical issue in designing interventions to reduce hospital use and improve community services is finding an optimal time point for post-discharge intervention. Although our sample size was relatively small, we observed that the gaps between admissions shortened over the first 3 admissions, especially for patients with bipolar disorder. Citrome et al. found that the time gap between multiple hospital stays of major depression disorders shortened over time, from 65 days between the 1st & 2nd stays to 24 days between the 5th & 6th stays [14]. The time gap itself and this shortening trend may provide significant reference on the design of community level mental health care programs in Beijing. This measure can also help further studies to analyze the time gaps with prospective patient follow-up data so that the factors influencing the time intervals between readmission can be studied.

### Possible approaches to support the secondary and community service

We found that the patients in the permanent stay cluster on average spent 1063/1095 days in tertiary hospitals and the average expenses on psychiatric inpatient services was 500,000 RMB (about 70,000 in US dollar) during the 3–year period. Secondary and community mental health programs should be developed or expanded to reduce the need and cost of long-term hospitalization in psychiatric patients [32, 41] .

In order to support the community-level efforts, tertiary hospitals also need to be incentivized to facilitate the patient transfer once certain criteria are met for a safe discharge. The Urban employee basic medical insurance (UEBMI) program in China currently adopted a per diem payment scheme for psychiatric patients (i.e. a fixed per diem fee is reimbursed regardless of the actual daily expenses or the LOS) [42–44]. Some have suggested this payment method may create financial incentive for the hospitals to extend the patients’ LOS [43]. As the daily expenses were likely to be decreasing over time for long-term stay patients [45], payment adjustment based on disease severity and patient LOS may help build a tiered psychiatric care system [42].

For patients in the permanent stay cluster, the average number of hospitalizations was 7.79 in the 3-year period even though they were hospitalized for virtually all the days in the 3-year observation period. This suggests that the discharge and admission process they underwent during the interim period were more likely for paperwork and/or reimbursement purposes than for an actual relapse or worsening of symptoms [48]. Same-day readmission may be a particular phenomenon in the Chinese inpatient care system and it deserves more attention, as the patients who need prolonged inpatient psychiatric care may benefit the most from alternative long-term care in the community. If all the permanent stay patients can be identified and transferred to secondary or community care, they would potentially free up about 40% of the hospital bed days for other patients who require acute inpatient care in tertiary hospitals. Therefore same-day readmission could serve as an indicator for the need of community-level service and guide the policies and programs to identify the target population for long-term care.

### Study limitations

Several limitations of this study need to be acknowledged. First, we only included patients from the 3 major tertiary psychiatric hospitals in Beijing, China. Since Beijing is the capital city of China and there are more psychiatric resources available than other places, our findings may not be generalizable to the entire country. However, the analytic methodology demonstrated in this study could be adopted for analysis of care in other regions.

Second, although we quantified the hospitalization expenses for the included patients, we were unable to break down the total expenses to analyze costs by day or for individual services. Nor could we determine out-of-pocket expenses for the patients. We also did not obtain the data on out-patient service use for the included patients. Therefore, the financial burden on the patients and their families may not be fully estimated in this study.

Third, since we adopted a rigorous set of patient inclusion criteria, patient characteristics should be taken into consideration when comparing our results with others. For example, we only included patients with no psychiatric admission records 3 years prior to the index admission, a criterion not often included in other studies.

## Conclusion

This study utilized the sequence analysis to characterize the psychiatric hospitalization trajectory, care utilization, and expenses for patients with 3 major psychiatric disorders in Beijing, China. Sequence analysis quantifies the concentration of high costs and patterns of frequent admissions among long-term and permanent stay patients and the results of this study strongly suggest that substantial needs for community-based treatment programs that would potentially reduce the psychiatric care costs and improve the quality of care for patients with severe psychiatric conditions. In the context of mental health care in China, insurance policies and health care financial schemes should also be structured to support these changes.

Since this study demonstrates how sequence analysis can be used to quantify psychiatric service utilization, the insights from this study and the adoption of these methods may be of value to countries or regions undergoing the “de-institutionalization” reform or trying to integrate mental health and non-psychiatric healthcare services.

Using these methods, future studies could also focus on identifying factors associated with the variation in hospitalization patterns and time gaps between multiple admissions for other chronic conditions. Such information may provide important references in the design and implementation of community programs to reduce inpatient service use and to move towards a primary care-based health care system.

## Supplementary Information

**Additional file 1.**

## Data Availability

The data used in this study was obtained from the inpatient medical record front page database of Beijing Municipal Health Commission Information Centre and was not publicly available. Contact information the Information Centre can be found on http://www.phic.org.cn/tjsj/.
