# Supplementary material for: A sequence analysis of hospitalization patterns and service utilization in patients with major psychiatric disorders in China
Source: BMC Psychiatry. 2021 May 11;21:245. doi: 10.1186/s12888-021-03251-w (PMC8111895; doi:10.1186/s12888-021-03251-w)
Supplement: Supplementary file 1 — Additional file 1. [file 12888_2021_3251_MOESM1_ESM.docx]

**Title page**

**Title**

**A sequence analysis of hospitalization patterns and service utilization in patients with major psychiatric disorders in China**

Xueyan Han^1^*, Feng Jiang^2^, Jack Needleman^3^, Moning Guo^4^, Yin Chen^5^, Huixuan Zhou^6^, Yuanli Liu^7^, Chen Yao^1,8^, Yilang Tang^9,10^

^1^ Peking University First Hospital, Beijing, China

^2^ Institute of Health Yangtze River Delta, Shanghai Jiao Tong University, Shanghai, China

^3^ Department of Health Policy and Management, UCLA Fielding School of Public Health, Los Angeles, California, USA

^4^ Beijing Municipal Health Commission Information Centre, Beijing, China

^5^ Peking University International Hospital, Beijing, China

^6^ School of Sport Science, Beijing Sport University, Beijing, China

^7^ School of public health, Chinese Academy of Medical Sciences and Peking Union Medical College, Beijing, China

^8^ Peking University Clinical Research Institute, Beijing, China

^9^ Department of Psychiatry and Behavioral Sciences, Emory University, Atlanta, Georgia, USA

^10^ Atlanta VA Medical Center, Decatur, Georgia, USA

*Corresponding author: Xueyan Han, Peking University First Hospital, 8 Xishiku Road, Xicheng District, Beijing, China, [hanxueyan611@163.com](mailto:hanxueyan611@163.com)

**Full names, institutional addresses and email addresses for all authors:**

Xueyan Han, Peking University First Hospital, 8 Xishiku Road, Xicheng District, Beijing, China, [hanxueyan611@163.com](mailto:hanxueyan611@163.com)

Feng Jiang, Institute of Health Yangtze River Delta, Shanghai Jiao Tong University, 1954 Huashan Road, Xuhui District, Shanghai, China, fengjiang@sjtu.edu.cn

Jack Needleman, Department of Health Policy and Management, UCLA Fielding School of Public Health, 650 Charles Young Dr. S., 31-269 CHS Box 951772, Los Angeles, CA, USA, needlema@ucla.edu

Moning Guo, Beijing Municipal Health Commission Information Centre, 277 Zhao Deng Yu Road, Xicheng District, Beijing, China, 13581627289@163.com

Yin Chen, Peking University International Hospital, 29 Sheng Ming Yuan Road, Haidian District, Beijing, China, chenyin@pkuih.edu.cn

Huixuan Zhou, School of Sport Science, Beijing Sport University, 48 Xinxi Road, Haidian Street, Beijing, China, [chouhuixuan@live.cn](mailto:chouhuixuan@live.cn)

Yuanli Liu, School of public health, Chinese Academy of Medical Sciences and Peking Union Medical College, No.3 Dong Dan San Tiao, Dongcheng District, Beijing, China, [liuyl_fpo@126.com](mailto:liuyl_fpo@126.com)

Chen Yao, Peking University First Hospital, 8 Xishiku Road, Xicheng District, Beijing, China; Peking University Clinical Research Institute, 38 Xueyuan Road, Haidian District Beijing, China, [yaochen.pucri@foxmail.com](mailto:yaochen.pucri@foxmail.com)

Yilang Tang, Department of Psychiatry and Behavioral Sciences, Emory University, 12 Executive Park Drive NE, Suite 300, Atlanta, GA, USA; Atlanta VA Medical Center, 1670 Clairmont Road, Decatur, GA, USA, [ytang5@emory.edu](mailto:ytang5@emory.edu)

**Manuscript title:** A sequence analysis of hospitalization patterns and service utilization in patients with major psychiatric disorders in China

**Additional File 1:**

**Supplementary material to the Methods section**

**1. Introduction on the “Secondary” and “tertiary” hospitals in China**

China has a 3-tiered hospital accreditation system, where hospitals are evaluated periodically according to the “Accreditation Criteria” issued by the government. The National Health Commission (NHC) and the provincial Health Commissions would grant the hospital with tertiary, secondary or primary status based on several measures of “hospital infrastructure, staffing, size and clinical capacity” [1]. According to the National Health Statistics Yearbook, only 7.8% (2548/33009) of all the hospitals in China were accredited as tertiary hospitals in 2018. The secondary hospitals accounted for 27.3% of all hospitals in 2018.

This 3-tiered hospital accreditation system is in correspondence with the hierarchical medical system of China, where the ideal scenario is for patients to seek help in primary healthcare facilities first and then moving up the tiers as the difficulty of treating their illnesses increases. However, the tertiary hospitals are mostly large in scale and may receive more funding from the government. They are also assumed to provide higher quality care compared with secondary hospitals. Compared with primary and secondary healthcare institutions, tertiary hospitals tended to have more experienced medical professionals and advanced equipment. Therefore, patients in China tended to bypass the primary and secondary healthcare and go directly to tertiary hospitals in almost all disease areas [2], not just for psychiatric patients.

This context explained why it would be of particular importance to investigate the difference in hospitalization pattern across the 3 tertiary psychiatric hospitals. Among specialty psychiatric hospitals, tertiary specialty psychiatric hospitals were much larger in size and treated more than 80% of the psychiatric inpatients in Beijing [3]. In this study we only included patients whose index psychiatric hospitalization was at one of the 3 tertiary psychiatric hospitals in Beijing.

**2. Psychiatric care resources in Beijing**

Apart from the 3-tiered hospital accreditation system, hospitals in China can also be divided into general and specialty hospitals. Although some general hospitals in Beijing did provide outpatient psychiatric services, inpatient psychiatric service (especially for patients with psychiatric disorders as their primary diagnoses) was almost exclusively provided by psychiatric hospitals. This was because 1) almost all the mental health professionals in China worked in specialty psychiatric hospitals; 2) mental health services provided by general hospitals are not very comprehensive [4].

In terms of the psychiatric care resources in Beijing, there are 14 secondary and tertiary psychiatric hospitals in Beijing and the database used by this study covered 10 out of these 14 hospitals (71.43%). The 10 hospitals consisted of 3 tertiary and 7 secondary psychiatric hospitals. Although the database did not cover all the psychiatric hospitals in Beijing we did include all the tertiary psychiatric hospitals in the city, which should cover a substantial portion of the regional psychiatric services.

The separation in the service provision (psychiatric hospitals provided mental health service but not much general healthcare while the general hospitals provided general healthcare and very limited mental healthcare service) promoted this study in examining the non-psychiatric service use (most likely happened in general hospitals). This context also stressed the importance of using a regional, multicenter database that included both psychiatric and general hospitals so that the patients’ inpatient service utilization can be better tracked and understood.

In order to understand the hospital-level difference of inpatient service use, this study included patients whose index psychiatric hospitalization was at one of the 3 tertiary psychiatric hospitals in Beijing (For full inclusion criteria please see part 2.2 of the main text). The 3 psychiatric hospitals were coded as hospital 1, 2, and 3 in the results session to represent each hospital and their differences.

**3. The Charlson comorbidity index and its utilization in this paper**

The Charlson comorbidity index (CCI) was originally developed by Charlson et al. in 1987. It was an index that included 19 categories of comorbidity and each patient would have a single comorbidity score to indicate their comorbidity status. The CCI was originally created to predict one-year mortality but was then used to indicate comorbidities in a range of studies [5, 6]. Sprah et al 2017 systematically reviewed the association between psychiatric readmissions and their association with physical comorbidity, and found that CCI was associated with increased risk of readmission in patients with substance use disorders [7]. Han et al. found that an increased number of comorbidities as measured by the Elixhauser comorbidity index was associated with both 30-day and 1-year readmission risk for psychiatric inpatients [3]. Since readmission is a key component of the long-term inpatient service utilization and the CCI has proved to be an influence factor of psychiatric readmission, this study included CCI as one of the factors to profile the patient characteristics in each hospitalization pattern.

The CCI version used in this study was published in Quan et al 2005 [8], which contained 17 categories of comorbidities (Supplementary table 1). It was the basis of a Stata module to calculate Charlson index of comorbidity (“CHARLSON”), which was used in this study. The CCI presented in this study was not weighted but a count of comorbidities listed in the CCI.

**4. The healthcare insurance system in China**

China is working towards achieving universal health coverage and have been expanding its health insurance coverage to all residents [9]. There are two social health insurance schemes for urban residents—the Urban Employee Basic Medical Insurance (UEBMI; launched in 1998) and the Urban Resident Basic Medical Insurance (URBMI; launched in 2007) [10]. There is also the New Cooperative medical scheme (NCMS) for rural residents. There are also a variety of other payment methods that existed like the private medical insurances but UEBMI, URBMI and NCMS were the 3 main insurance types in China. Since 2016, URBMI and NCMS have been combined as Basic medical insurance for urban and rural residents. Hence, we classified the payment methods in this paper as UEBMI, other insurances and uninsured.

Although the coverage of these social insurances is getting higher, different social insurance types do have different funding sources and therefore different benefit packages [10]. Xu et al had found that insurance types and reimbursement ratios can have significant association with the utilization of inpatient services. In this study we also included insurance types as one of the factors to profile the patient characteristics in each hospitalization pattern [11].

| **Supplementary table 1. The** **ICD-10 Codes for the Charlson Comorbidities** | |
| --- | --- |
| **Comorbidities** | **ICD-10** |
| Myocardial infarction | I21.x, I22.x, I25.2 |
| Congestive heart failure | I09.9,I11.0, I13.0, I13.2, I25.5, I42.0, I42.5-I42.9, I43.x, I50.x, P29.0 |
| Peripheral vascular disease | I70.x, I71.x, I73.1, I73.8, I73.9, I77.1, I79.0, I79.2, K55.1, K55.8, K55.9, Z95.8, Z95.9 |
| Cerebrovascular disease | G45.x, G46.x, H34.0, I60.x-I69.x |
| Dementia | F00.x-F03.x, F05.1, G30.x, G31.1 |
| Chronic pulmonary disease | I27.8, I27.9, J40.x-J47.x, J60.x-J67.x, J68.4, J70.1, J70.3 |
| Rheumatic disease | M05.x, M06.x, M31.5, M32.x-M34.x, M35.1, M35.3, M36.0 |
| Peptic ulcer disease | K25.x-K28.x |
| Mild liver disease | B18.x, K70.0-K70.3, K70.9, K71.3-K71.5, K71.7, K73.x, K74.x, K76.0, K76.2-K76.4, K76.8, K76.9, Z94.4 |
| Diabetes without chronic complication | E10.0, E10.l, E10.6, E10.8, E10.9, E11.0, E11.1, E11.6, E11.8, E11.9, E12.0, E12.1, E12.6, E12.8, E12.9, E13.0, E13.1, E13.6, E13.8, E13.9, E14.0, E14.1, E14.6, E14.8, E14.9 |
| Diabetes with chronic complication | E10.2-E10.5, E10.7, E11.2-E11.5, E11.7, E12.2-E12.5, E12.7, E13.2-E13.5, E13.7, E14.2-E14.5, E14.7 |
| Hemiplegia or paraplegia | G04.1, G11.4, G80.1, G80.2, G81.x, G82.x, G83.0-G83.4, G83.9 |
| Renal disease | I12.0, I13.1, N03.2-N03.7, N05.2-N05.7, N18.x, N19.x, N25.0, Z49.0-Z49.2, Z94.0, Z99.2 |
| Any malignancy, including lymphoma and leukemia, except malignant neoplasm of skin | C00.x-C26.x, C30.x-C34.x, C37.x-C41.x, C43.x, C45.x-C58.x, C60.x-C76.x, C81.x-C85.x, C88.x, C90.x-C97.x |
| Moderate or severe liver disease | I85.0, I85.9, I86.4, I98.2, K70.4, K71.1, K72.1, K72.9, K76.5, K76.6, K76.7 |
| Metastatic solid tumor | C77.x-C80.x |
| AIDS/HIV | B20.x-B22.x, B24.x |

**5. Further clarifications on data cleansing**

After patients were identified, their index admission in 2013 and any subsequent hospitalization records for either psychiatric or non-psychiatric conditions were identified using the unique identification code of each patient. Since the observation period was 3 years after the index admission date, Day-1 in this study would be the index admission date for each patient, and Day-1095 would be the last day of follow-up. Admissions after Day-1095 were excluded. For patient records with admission dates before Day-1095 and discharge date after Day-1095, the length of stay (LOS) of that hospitalization was calculated from the admission date to Day-1095. For these admissions, we adjusted the included expenses of that inpatient stay: adjusted expenses=total expenses*(LOS within the 1095 day period/ total LOS). For the patients who appeared to be readmitted on the same day of previous discharge, perhaps due to a scheduled transfer, we reduced by 1 day the LOS of the latter admission.

**6. Details regarding the sequence analysis method used in this study**

This study used a combination of state-sequence analysis and the cluster analysis to capture the inpatient service utilization in the included psychiatric patients.

The analyses were conducted using the TraMineR package in R (version 3.4.4) [12]. The package enables discrete sequential-state analysis on a sequence of event or state.

Essentially, this method has 4 steps: 1) data transformation and hospitalization sequence building; 2) Analyzing the sequences with the optimal matching algorithm which forms a dissimilarity matrix; 3) Applying cluster analysis on the dissimilarity matrix; 4) Determining the number of clusters and summarizing the hospitalization pattern for patients included in each cluster. Sequences in each identified cluster were visualized. This method has been described and used in Han et al, 2020 [13] and Golay et al, 2019 [14].

**Step 1**: A state in the state-sequence analysis means the status of the patients (e.g. admitted, not admitted), and the state can be represented by numbers. For each patient, their admissions were aggregated into a string that contained 1095 digits, each digit represented one in the 1095 days (3 years) of observation [13, 14]. The digit was determined by the status of the patients, and there were 2 options (states): 0-not admitted, 1-admitted for major psychiatric disorders (MPD). For example, if a patient was admitted for MPD for 3 days, got discharged and then readmitted after 2 days of staying at home, the first few digits of the patient’s sequence would be: 111001…

After the data transformation, each patient would have the corresponding sequence to represent the hospitalization pattern in the 3-year period.

**Step 2**: The sequences were then analyzed using the optimal matching algorithm. Specifically, the dissimilarity of each pair of sequences was computed using the optimal matching algorithm which generates edit distances that are the minimal cost, in terms of insertions, deletions and substitutions, for transforming one sequence into another [15]. This step produced a dissimilarity matrix.

**Step 3**: A cluster analysis using the Ward’s method was then performed on this dissimilarity matrix to group patients with similar individual trajectories into clusters. It should be noted that the cluster analysis was not based on total hospital stay or the frequency of hospitalization alone, it was the cluster analysis on a dissimilarity matrix which would reflect the hospitalization sequence of each patient.

**Step 4**: The number of clusters were determined based on the dendrogram of cluster analysis and interpretability. The visualization tools in the TraMineR package were utilized to illustrate the hospitalization pattern of patients in each cluster.

**References**

1. Jian W, Figueroa J, Woskie L, Yao X, Zhou Y, Li Z, Li C, Yao L, Yip WC: **Quality of care in large Chinese hospitals: an observational study**. *BMJ QUAL SAF* 2019:2018-8938.

2. Liu G, Xue Y, Qian Z, Yang L, Yang Y, Geng Q, Wang X: **Healthcare-seeking behavior among pregnant women in the Chinese hierarchical medical system: a cross-sectional study**. *INT J EQUITY HEALTH* 2019, **18**(1).

3. Han X, Jiang F, Tang Y, Needleman J, Guo M, Chen Y, Zhou H, Liu Y: **Factors associated with 30-day and 1-year readmission among psychiatric inpatients in Beijing China: a retrospective, medical record-based analysis**. *BMC PSYCHIATRY* 2020, **20**(1).

4. Shi J, Tang L, Jing L, Geng J, Liu R, Luo L, Chen N, Liu Q, Gong X, Bo X *et al*: **Disparities in mental health care utilization among inpatients in various types of health institutions: a cross-sectional study based on EHR data in Shanghai, China**. *BMC PUBLIC HEALTH* 2019, **19**(1).

5. Setter NW, Peres ML, Almeida BMM, Petterle RR, Raboni SM: **Charlson comorbidity index scores and in‐hospital prognosis of patients with severe acute respiratory infections**. *INTERN MED J* 2020, **50**(6):691-697.

6. Lin Y, Yang C, Chu H, Wu J, Lin K, Shi Y, Wang H, Kong G, Zhang L: **Association between the Charlson Comorbidity Index and the risk of 30-day unplanned readmission in patients receiving maintenance dialysis**. *BMC NEPHROL* 2019, **20**(1):363.

7. Šprah L, Dernovšek MZ, Wahlbeck K, Haaramo P: **Psychiatric readmissions and their association with physical comorbidity: a systematic literature review**. *BMC PSYCHIATRY* 2017, **17**(1).

8. Quan H, Sundararajan V, Halfon P, Fong A, Burnand B, Luthi JC, Saunders LD, Beck CA, Feasby TE, Ghali WA: **Coding algorithms for defining comorbidities in ICD-9-CM and ICD-10 administrative data**. *MED CARE* 2005, **43**(11):1130-1139.

9. Tao W, Zeng Z, Dang H, Lu B, Chuong L, Yue D, Wen J, Zhao R, Li W, Kominski GF: **Towards universal health coverage: lessons from 10 years of healthcare reform in China**. *BMJ Global Health* 2020, **5**(3):e2086.

10. Zhang H, Sun Y, Zhang D, Zhang C, Chen G: **Direct medical costs for patients with schizophrenia: a 4-year cohort study from health insurance claims data in Guangzhou city, Southern China**. *INT J MENT HEALTH SY* 2018, **12**(1).

11. Xu J, Wang J, King M, Liu R, Yu F, Xing J, Su L, Lu M: **Rural–urban disparities in the utilization of mental health inpatient services in China: the role of health insurance**. *International Journal of Health Economics and Management* 2018, **18**(4):377-393.

12. Gabadinho A, Ritschard G, Müller NS, Studer M: **Analyzing and Visualizing State Sequences in R with TraMineR**. *J STAT SOFTW* 2011, **40**(4).

13. Han X, Jiang F, Zhou H, Needleman J, Guo M, Chen Y, Liu Y, Tang Y: **Hospitalization Pattern, Inpatient Service Utilization and Quality of Care in Patients With Alcohol Use Disorder: A Sequence Analysis of Discharge Medical Records**. *ALCOHOL ALCOHOLISM* 2020, **55**(2):179-186.

14. Golay P, Morandi S, Conus P, Bonsack C: **Identifying patterns in psychiatric hospital stays with statistical methods: towards a typology of post-deinstitutionalization hospitalization trajectories**. *SOC PSYCH PSYCH EPID* 2019, **54**(11):1411-1417.

15. Gabadinho A, Ritschard G, Studer M, Müller N: **Mining sequence data in R with the TraMineR package: A user's guide1**. University of Geneva, Geneva; 2009.
